# Enhanced oral bioavailability of cannabidiol by flexible zein nanoparticles: *in vitro* and pharmacokinetic studies

**DOI:** 10.3389/fnut.2024.1431620

**Published:** 2024-07-17

**Authors:** YingLan Nie, Yan Kong, Juan Peng, Jian Sun, Bin Fan

**Affiliations:** Beijing Key Laboratory of Basic Research on Traditional Chinese Medicine to Prevent and Control Major Diseases, Experimental Research Center, China Academy of Chinese Medical Sciences, Beijing, China

**Keywords:** flexible zein, cannabidiol, nanoparticle, bioavailability, cannabidiol release

## Abstract

**Introduction:**

Cannabidiol (CBD) has a variety of pharmacological effects including antiepileptic, antispasmodic, anxiolytic and anti-inflammatory among other pharmacological effects. However, since CBD is a terpene-phenolic compound, its clinical application is limited by its poor water solubility, low stability, and low bioavailability.

**Methods:**

In this study, we used several strategies to address the above problems. Hydrochloric acid was used to modify zein to improve the molecular flexibility. Flexible zein nanoparticles (FZP-CBD) loaded with CBD was prepared to improve the stability and bioavailability of CBD. The parameters were evaluated in terms of morphology, particle size (PS), polydispersity index (PDI), zeta potential (ZP), entrapment efficiency (EE%), loading capacity (LC%), and storage stability. Simulated gastrointestinal fluid release experiment and bioavailability assay were applied in the evaluation.

**Results:**

The simulated gastrointestinal fluid experiment showed that the release rates of FZP-CBD and natural zein nanoparticles (NZP-CBD) loaded with CBD were 3.57% and 89.88%, respectively, after digestion with gastric fluid for 2 h, 92.12% and 92.56%, respectively, after intestinal fluid digestion for 2 h. Compared with NZP-CBD, the *C*_max_ of FZP-CBD at 3 different doses of CBD was increased by 1.7, 1.3 and 1.5 times respectively, and AUC_0-t_ was increased by 1.4, 1.1 and 1.7 times respectively, bioavailability (F) was increased by 135.9%, 114.9%, 169.6% respectively.

**Discussion:**

The experimental results showed that FZP-CBD could protect most of the CBD from being released in the stomach, and then control its release in the intestines, promote the absorption of CBD in the small intestine, and increase the bioavailability of CBD. Therefore, FZP-CBD could improve the utilization value of CBD and provide a new idea for the application of CBD in medicine and pharmacy.

## 1 Introduction

Cannabidiol (CBD) is one of the main extracts of *Cannabis sativa* L., an annual herb of the family Moraceae. Currently, more than 130 cannabinoids have been isolated from dried plants and fresh cannabis leaves. Among them, the tetrahydrocannabinol (THC) and CBD contents are the highest. While THC is hallucinogenic, CBD is a non-addictive component because it can antagonize the psychoactivity triggered by THC agonism at cannabinoid receptor I (CB1R). CBD has antiepileptic, antispasmodic, anxiolytic and anti-inflammatory effects among other pharmacological effects (1, 2). However, CBD is a terpene phenolic compound. It is highly lipophilic (logP = 6.3) with a low solubility in water of 0.02–0.06 μg•mL^−1^ at room temperature (3). Currently approved CBD drugs are mostly ethanol or oil-based (4), such as Epidiolex^®^ (a pure CBD oral solution suspended in sesame seed oil and ethanol) and Sativex^®^ (a CBD and Delta-9-tetrahydrocannabinol (THC, 1:1) oromucosal spray suspended in anhydrous ethanol and propylene glycol) (5, 6) which were approved by the United States (US) Food and Drug Administration and the European Medicine Agency, respectively. However, highly lipophilic drugs delivered orally tends to precipitate in the gastrointestinal (GI) tract and are difficult to be completely solubilized. This is the main reason for its low bioavailability (7). Some studies have shown that the oral bioavailability of CBD oil in human trials is about 6% (8–10). CBD is mainly absorbed in the upper part of the small intestine (11). CBD has poor water solubility and stability, and it may be inactivated due to degradation, especially under acid, oxygen, high temperature, light radiation and other conditions, thus significantly reducing its bioavailability and utilization value (12). To improve the bioavailability and therapeutic efficacy of CBD, our study aims to develop a safe dosage form for the delivery of CBD that not only reduces metabolic degradation but also improves its solubility.

Nanodelivery system is a technology to deliver drugs or other biomolecules to specific cells or tissues through a carrier called nanoparticles. Mucoadhesion of nanoparticles depends on the presence of mucoadhesive polymers. Once properly selected, the efficiency of adhesion and interaction with cells may improve due to their size and large specific surface area. They can also improve the poor solubility of hydrophobic drugs, low bioavailability and short half-life (13–15), thereby demonstrating significant promise in increasing the oral bioavailability of highly lipophilic compounds (12). Nanoparticles are small colloidal particles with three dimensions in nanomaterials. Protein particles are ubiquitous in the field of drug delivery. They have intrinsic bioactivity, biocompatibility, biodegradability and colloidal properties, including self-assembly behavior, structural reorganization, intermolecular and intramolecular hydrophobic and hydrogen bonding interactions (16). Nanoparticles can be absorbed in organisms by two routes. The first is to be directly taken up by small intestinal epithelial cells via inducing endocytosis (14). For example, the presence of lactoferrin receptors in intestinal epithelial cells can help lactoferrin nanoparticles containing *Garcinia cambogia* to cross the epithelial cells into the blood vessels (17). To achieve endocytosis, the nanoparticles must be small in size and their surface properties must be carefully designed to facilitate cell entry (18, 19). Larger particles or particles with highly dense cationic surfaces may induce the formation of pores on membranes and result in cytotoxicity (18). Another way of absorption is that the nanoparticles can be digested by digestive enzymes in the gastrointestinal tract, which frees the active substance and transports it into the blood circulation. The active substance is absorbed through two main transport mechanisms, active and passive transport in the small intestine (13).

Zein is an important storage protein in corn (20). It contains more than half of its amino acids in a nonpolar form, lacks charged amino acids, and is low in polar amino acids. As a result, it is not soluble in pure water and anhydrous alcoholic media. However, it can be dissolved into 50–95% aqueous ethanol solutions (21). Zein is subject to reduced solubility due to decreased ethanol concentration and increased solvent polarity, which causes a change in protein conformation in the process, resulting in aggregation of the molecules, a process known as self-assembly of zein (21). Zein can be induced to self-assemble into different nanostructures such as nanoparticles, micelles, fibers and membranes by adjusting the polarity of the medium (22). The anti-solvent method is one of the commonly used methods for the preparation of zein nanoparticles. In this method, zein undergoes self-assembly and aggregates into nanoparticles to evade highly polar environments when the polarity of the medium changes from weak to strong (23). Zein nanoparticles have been used as nanocarrier materials in the aspects of encapsulation efficiency, stability and drug release (22, 24–26). However, due to the strong hydrophobicity and rigid structure of zein, the nanoparticles formed by natural zein are poorly adapted to the environment. Therefore, they are not perfect for drug delivery. To improve the colloidal stability of zein particles or to regulate the drug release rate, many researchers have attempted to improve the EE% or control the drug release by introducing other polymers or using acid to modify zein nanoparticles (27, 28), and subsequently improving the ability of zein nanoparticles to transport small molecule drugs. For example, Dai et al. improved the entrapment efficiency (EE%) and stability of zein nanoparticles to hydrophobic drugs by adding rhamnose (29). Van Ballegooie et al. modified zein with polyethylene glycol (PEG) to improve its stability (30). Ahammed et al. used glutamine-transaminase (TG) modified gelatin-zein complex loaded with green tea polyphenols (TP) to control the release of phenolic compounds (31). In this experiment, we prepared nanoparticles to properly encapsulate and transport CBD by enhancing the flexibility of zein, facilitating its delivery of CBD to the small intestine for release and absorption. This approach can improve the utilization efficiency of CBD to a certain extent.

In this study, flexible zein was obtained by acid modification of zein and flexible zein nanoparticles (FZP-CBD) and natural zein nanoparticles (NZP-CBD) loaded with CBD were prepared. We Studied their morphology, particle size (PS), polydispersity index (PDI), Zeta potential (ZP), EE%, loading capacity (LC%), storage stability, and *in vitro* simulated gastrointestinal fluid release. The oral bioavailability of the two was also explored for comparison by *in vivo* pharmacokinetic experiments in rats. This study improves the value of CBD utilization and broadens the scope of zein application.

## 2 Materials and methods

### 2.1 Materials and instruments

Agilent 6,410 QQQ LC–MS analysis system, consisting of an Agilent 1,290 liquid chromatograph and Agilent 6,410 QQQ triple quadrupole mass spectrometry detector, Masshunter workstation was obtained from Agilent Technologies. Hettich Mikro 220R benchtop cryogenic high-speed centrifuge was obtained from Hettich. Gene G560E vortex mixer was obtained from Scientific Industries. Milli-Q Integral 3 integrated ultrapure water system was obtained from Merck KGaA. Sartorius SQP QUINTIX65-1CN one-millionth electronic balance was obtained from Sartorius. DK-98-II electrothermal thermostatic water bath was obtained from Tianjin Test Instrument Co., Ltd. PH meter was obtained from Sartorius. V-850 rotary evaporator was obtained from BUCHI. Hitachi S-4800 field emission scanning electron microscope was obtained from Hitachi. LC-250 Nanoparticle Size and Zeta Potential Analyzer was obtained from Microtrac. SW-CJ-2FD Cell Incubator was obtained from Thermo Fisher Scientific.

Zein was obtained from Yuan Ye Biotechnology Co., Ltd. Cannabidiol (CBD) was obtained from Heilongjiang Zhongsheng Bio-technology Co., Ltd. (−)-Cannabidiol-d9 (internal standard) was obtained from Shanghai Saiers Biochemical Technology Co., Ltd. Methanol for chromatography, Acetonitrile for chromatography and Formic acid for chromatography were obtained from Dikma. Anhydrous ethanol (analytically pure) and hydrochloric acid (analytically pure) were obtained from Sinopharm Chemical Reagent Co., Ltd. Sodium hydroxide (analytically pure) was obtained from Beijing Chemical Industry Co., Ltd. Potassium Dihydrogen Phosphate, pepsin and trypsin were obtained from Source Leaf Biotechnology Co., Ltd. and Ultra-pure water (self-made).

Rat adrenal pheochromocytoma cell lines (PC12 cells) were obtained from the Cell Resource Center, Shanghai Academy of Biological Sciences, Chinese Academy of Sciences.

There were 36 Sprague Dawley (SD) rats aged 7–8 weeks (half male and half female), weighing 220–300 g purchased from Beijing Huafukang Bio-technology Co., Ltd. NO. 110322230100338248. All rats were fed in SPF level animal room. All animal experiments were approved by the animal ethics committee of Experimental Research Center, China Academy of Chinese Medical Sciences (ERCCACMS21-2211-01).

### 2.2 Methods

#### 2.2.1 Preparation of nanoparticles

Zein alcohol solution loaded with CBD: zein was fully dispersed in 90% ethanol to obtain 25 mg•mL^−1^ of zein ethanol solution. The solution was equally divided into two groups. One group was left untreated and the other was adjusted to pH 2.0 with HCl and then heated by condensation reflux in a water bath at 70°C for 10 h to obtain a flexible zein alcohol solution. NaOH solution was used to adjust the pH of the flexible zein alcohol solution to 7.0. Both groups of solutions were divided into three equal portions, then different amounts of CBD were added, respectively, to obtain zein alcohol solution containing CBD with low, medium and high concentrations (4.10 and 16 mg•mL^−1^).

FZP-CBD and NZP-CBD: FZP-CBD and NZP-CBD were prepared by rapid anti-solvent precipitation method, in which the zein alcohol solution loaded with CBD was rapidly poured into ultrapure water at a volume ratio of 1:4 while stirring with 40°C. The ethanol was removed by rotary evaporation, and then ultrapure water was added to replenish the volume of the ethanol that had been lost. NZP-CBD and FZP-CBD loaded with varying amounts of CBD were obtained. They were stored in colloidal solution form at 4°C in the refrigerator for future use.

#### 2.2.2 Morphology

The microscopic morphology of NZP-CBD and FZP-CBD was observed using scanning electron microscopy (SEM) at an operating voltage of 5 kV and a magnification of 45,000×. A small amount of freshly prepared samples was applied to silicon wafers, which were naturally dried and then sprayed with gold, imaged and photographed.

#### 2.2.3 PS, PDI, and ZP

The average PS and PDI of NZP-CBD and FZP-CBD were measured by the dynamic light scattering (DLS) method by using the Nano-ZS nanolaser, and the potential at the nanoparticle shear plane, denoted as the ZP, was measured using the above instrument.

#### 2.2.4 EE% and LC%

High-performance liquid chromatography (HPLC) was used to determine the EE% and LC% of the nanoparticles.

##### 2.2.4.1 Establishment of HPLC methods

The chromatographic conditions were as follows: chromatographic column Diamonsil C18 (2) (250 mm × 4.6 mm, 5 μm); mobile phase methanol–water (87:13, v/v); flow rate 1.0 mL•min^−1^; detection wavelength 220 nm; and column temperature 25°C.

The EE% and LC% of FZP-CBD and NZP-CBD were calculated using the following Equations (1) and (2):


(1) $$EE\left(\%\right)=\frac{C_{\text{system}}}{C_{\text{system}}+C_{\text{free}}}\times 100$$

(2) $$LC\left(\%\right)=\frac{W_{\text{system}}}{W_{\text{total}}}\times 100$$

*C*_system_ and *C*_free_ are the concentrations of encapsulated CBD and free CBD in the colloidal system, respectively. *W*_system_ and *W*_total_ are the mass of the encapsulated CBD and the total mass of the nanoparticles in the colloidal system, respectively.

##### 2.2.4.2 Free CBD extraction

We took 100 μL of different concentrations of NZP-CBD and FZP-CBD and added 300 μL of petroleum ether to extract three times. Then it was redissolved by adding 1 mL of acetonitrile after nitrogen blowing, vortexed for 30 s, and injected the sample.

##### 2.2.4.3 Encapsulated CBD extraction

After drying under nitrogen, the nanoparticles were extracted with 900 μL acetonitrile, vortexed for 2 min at 16,000 r•min^−1^, 4°C, centrifuged for 10 min, 100 μL supernatant was mixed with 900 μL acetonitrile, vortexed for 30 s, and injected into the column.

#### 2.2.5 Storage stability

The prepared NZP-CBD and FZP-CBD were stored at 4°C under light-avoidance conditions, and the degradation of CBD was measured at day 0, 3, 7, 14, and 21. The storage stability of NZP-CBD and FZP-CBD was expressed by the Leaching Rate and it was calculated using the following Equation (3).


(3) $$\text{Leaching}\;\text{Rate}=\frac{W_{\text{free}}}{W_{\text{system}}+W_{\text{free}}}\times 100$$

where *W*_system_ and *W*_free_ are the mass of encapsulated CBD and the mass of degraded CBD in the colloidal system, respectively.

#### 2.2.6 Simulated gastrointestinal fluid release

Ten milliliter of prepared NZP-CBD and FZP-CBD suspensions were mixed with 10 mL of simulated gastric fluid (SGF) containing 10 mg•mL^−1^ pepsin at pH 2 and then incubated for 2 h (samples were collected at 0, 0.5, 1, 1.5, 2 h) in a shaker at 37°C with shaking at a speed of 100 rpm. After 2 h of digestion, the pH was adjusted to 7.5 using NaOH solution to inactivate the enzyme. Subsequently, 10 mL digestion mixture was poured into the 10 mL of simulated intestinal fluid (SIF, dissolving pancreatin (10 mg•mL^−1^) in phosphate buffer at pH 6.8.) and incubated for 2 h (samples were collected at 0, 0.5, 1, 1.5, 2 h) under the same conditions and then terminated by rapid cooling in ice. The CBD was determined by HPLC and its digestion was expressed by Release Rate (Eq. 4).


(4) $$\text{Release}\;\text{Rate}=\frac{W_{1}}{W_{0}}\times 100$$

Where *W*_1_ is the amount of CBD released and *W*_0_ is the amount of CBD wrapped at 0 h.

#### 2.2.7 *In vitro* biocompatibility

To investigate the biocompatibility of FZP-CBD, the experiments used PC12 cells as a cell model. The cytotoxicity was detected by CCK8. Logarithmic growing PC12 cells were inoculated into 96-well cell culture plates at a density of 8 × 10^4^ cells•mL^−1^ per well, and cultured in a 5% CO_2_ cell incubator at 37°C. When the cell density reached to 60%, different concentrations of CBD solution and FZP-CBD (0, 1, 2.5, 5, 10, 15, 20, 25 μM) were added to PC12 cells after being completely diluted with DMEM medium to continue the incubation for 24 h. Then 10% CCK8 staining solution was added to each well and continued culturing for 1.5 h. Absorbance (OD value) at 490 nm wavelength was measured by BioTek Synergy H1 Hybrid multimode microplate reader. Taking the blank nanoparticles without CBD as a reference, the experiment was repeated 3 times, and cell viability was calculated using the following Equation (5).


(5) $$\text{Viability}\left(\%\right)=\frac{A-A_{1}}{A_{0}-A_{1}}\times 100$$

Where *A* is the OD value of the cells in the sample wells, *A*_0_ is the OD value of the cells in the blank group, and *A*_1_ is the OD value of the blank wells.

#### 2.2.8 *In vivo* bioavailability

36 SD rats were divided into six groups. NZP-CBD and FZP-CBD in 3 doses of 20, 50 and 100 mg·kg^−1^, respectively, were administrated intragastric in each group. Blood samples were collected from the oculi chorioideae vein at 0, 0.5, 1, 1.5, 2, 3, 4, 6, 8, 10, 12, 24, and 48 h after the administration. Blood samples were centrifuged at 3,000 rpm·min^−1^ for 10 min at 4°C. The supernatant was stored at −80°C for measurement.

Supernatant (20 μL) was added to 60 μL of methanol and centrifuged at 12,000 rpm•min^−1^ for 10 min at 4°C. The supernatant was extracted and analyzed by LC–MS. The pharmacokinetic parameters, including half-life (*T*_1/2_), peak time (*T*_max_), peak concentration (*C*_max_), area under the curve (AUC), and mean residence time (MRT) were statistically analyzed by Winnonlin 8.1.

Chromatographic conditions: column: Waters Atlantis T3 (100 mm × 2.1 mm, 3 μm), mobile phases: A is 0.1% formic acid-water, B is 0.1% formic acid-acetonitrile, gradient elution, 0.0–4.5 min, 85% B, 4.5–5.0 min, 85–100% B, column temperature 30°C, flow rate 0.3 mL•min^−1^, injection volume 1 μL.

Mass spectrometry conditions: electrospray ionization source (ESI), multiple reaction monitoring (MRM), positive ion mode detection, CBD ion pair 315.3 → 259.3, isotopic internal standard (−)-Cannabidiol-d9 ion pair 324 → 268.2, collision energy CBD is 15 V, the internal standard is 20 V, fragment 110 V for CBD and 170 V for the internal standard, atomizing gas pressure 15 psi, ion source temperature 300°C, and atomizing gas flow rate 12 L•min^−1^.

## 3 Results and discussion

### 3.1 Morphology

**Figure 1:**
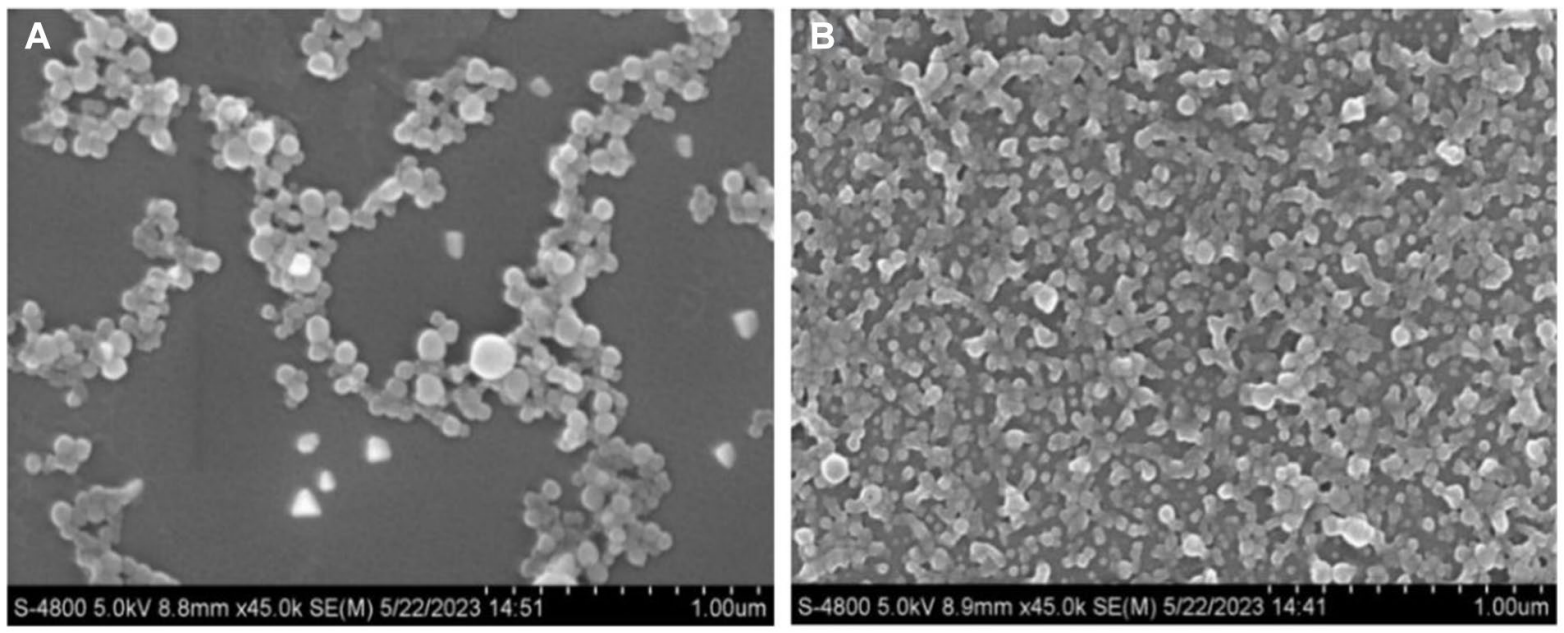
Micromorphology (magnification of 45,000×) of FZP-CBD **(A)** and NZP-CBD **(B)** loaded with CBD.

Dong (32) and Mattice and Marangoni (33) found that the percentage of the α-helical structure of zein decreased and intermolecular β-folding increased after being treated with aqueous acetic acid. This may be due to the change of α-helix to β-folding and irregularly curled structures in the protein, resulting in the protein becoming loose and disordered, which makes the protein structure flexible (34). The distribution of nanoparticles formed by natural zein is more dense and ordered, forming a reticular structure. In contrast, the nanoparticles formed by flexible zein are sparse and disordered, indicating the flexibility of this part of the protein structure (Figure 1). This facilitates the binding of zein to CBD and improves the storage stability of the drug.

### 3.2 PS, PDI, and ZP

**Figure 2:**
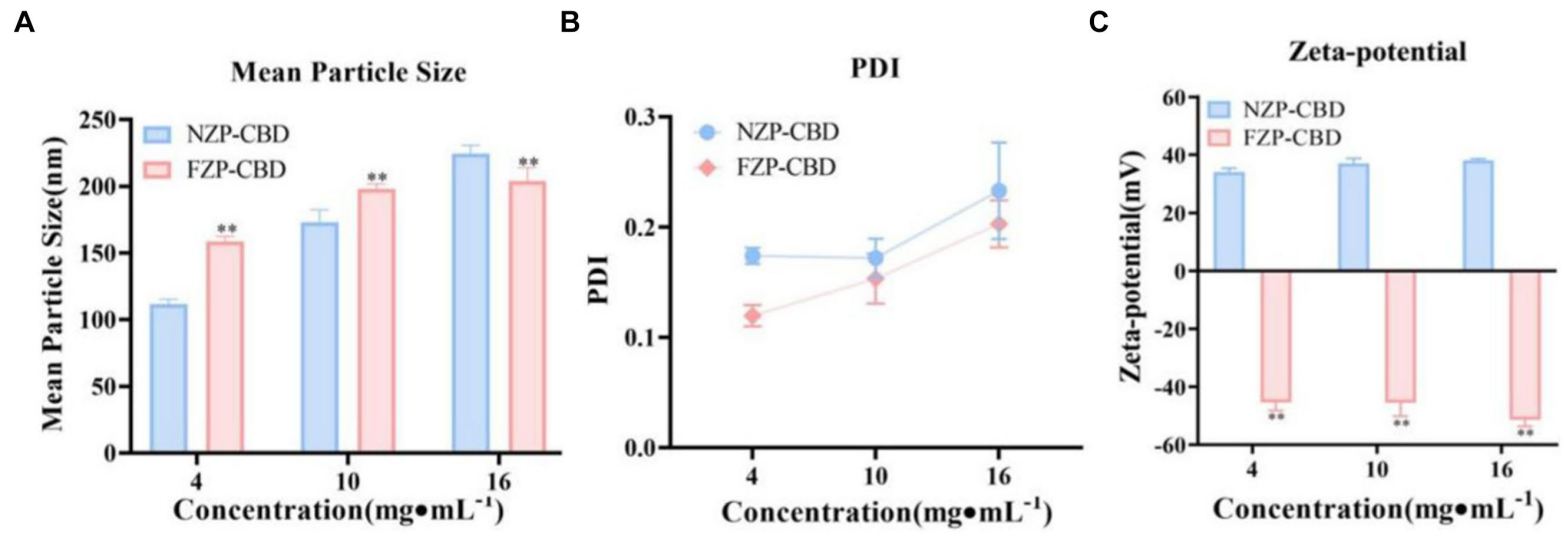
PS **(A)**, PDI **(B)**, and ZP **(C)** of NZP-CBD and FZP-CBD. ^*^p < 0.05, ^**^p < 0.01 compared to NZP-CBD (*x* ± s, *n* = 3).

The PS, PDI and ZP of NZP-CBD and FZP-CBD were shown in Figure 2. The results suggested that the larger PS of FZP-CBD may be the reason for the increased LC%. The PDI of FZP-CBD and NZP-CBD at low, medium and high concentrations were 0.12, 0.15, and 0.20 and 0.17, 0.17, and 0.23, respectively. This may be because FZP-CBD could prevent protein aggregation through electrostatic interactions (35), resulting in a more uniform distribution of proteins in solution with smaller PDI.

As shown in Figure 2C, due to deamidation, the amide group is converted to a carboxyl group, and the FZP-CBD’s ZP became negative after ionization, which was between −52 and −45 mV. The NZP-CBD’s ZP was between 34 and 38 mV due to the ionization of amide groups. The value of ZP is related to the stability of colloidal dispersion, with the absolute value representing the degree of stability (36). The greater the absolute value, the better the stability. The absolute value of FZP-CBD was significantly larger than that of NZP-CBD (*p* < 0.01), indicating that FZP-CBD is employed to enhance the dispersibility of proteins by substituting the amino group in amide residues with a carboxyl group (37), thereby increasing electrostatic repulsion to prevent the aggregation of particles and thus has higher stability (32, 35).

### 3.3 EE% and LC%

The CBD entrapped inside of the nanoparticles was determined using newly prepared colloidal solutions of NZP-CBD and FZP-CBD at low, medium and high concentrations (Supplementary Table S2). There were some differences between NZP-CBD and FZP-CBD. The EE% and LC% of CBD in NZP-CBD were 72.89, 62.11, 79.27, and 29.30%, 36.86, 53.63% for low, medium and high concentrations, respectively, while that of FZP-CBD was 92.23, 86.14, 85.43 and 34.68%, 48.86, 56.94% for low, medium and high concentrations, respectively. The deamidation reaction which was performed by acid modification converts amide groups to carboxyl groups, and destroys the helical structure, thus resulting in increased β-folding and decreased.

α-helix (32). This may induce the gradual unfolding and disordering of zein’s tertiary structure and increase the protein’s interfacial adsorption capacity (38). Thereby it is conducive to the adsorption and binding of zein and CBD. Therefore, the EE% and LC% of FZP-CBD were higher than that of NZP-CBD, and the EE% and LC% of CBD could be improved by FZP-CBD.

### 3.4 Storage stability

**Figure 3:**
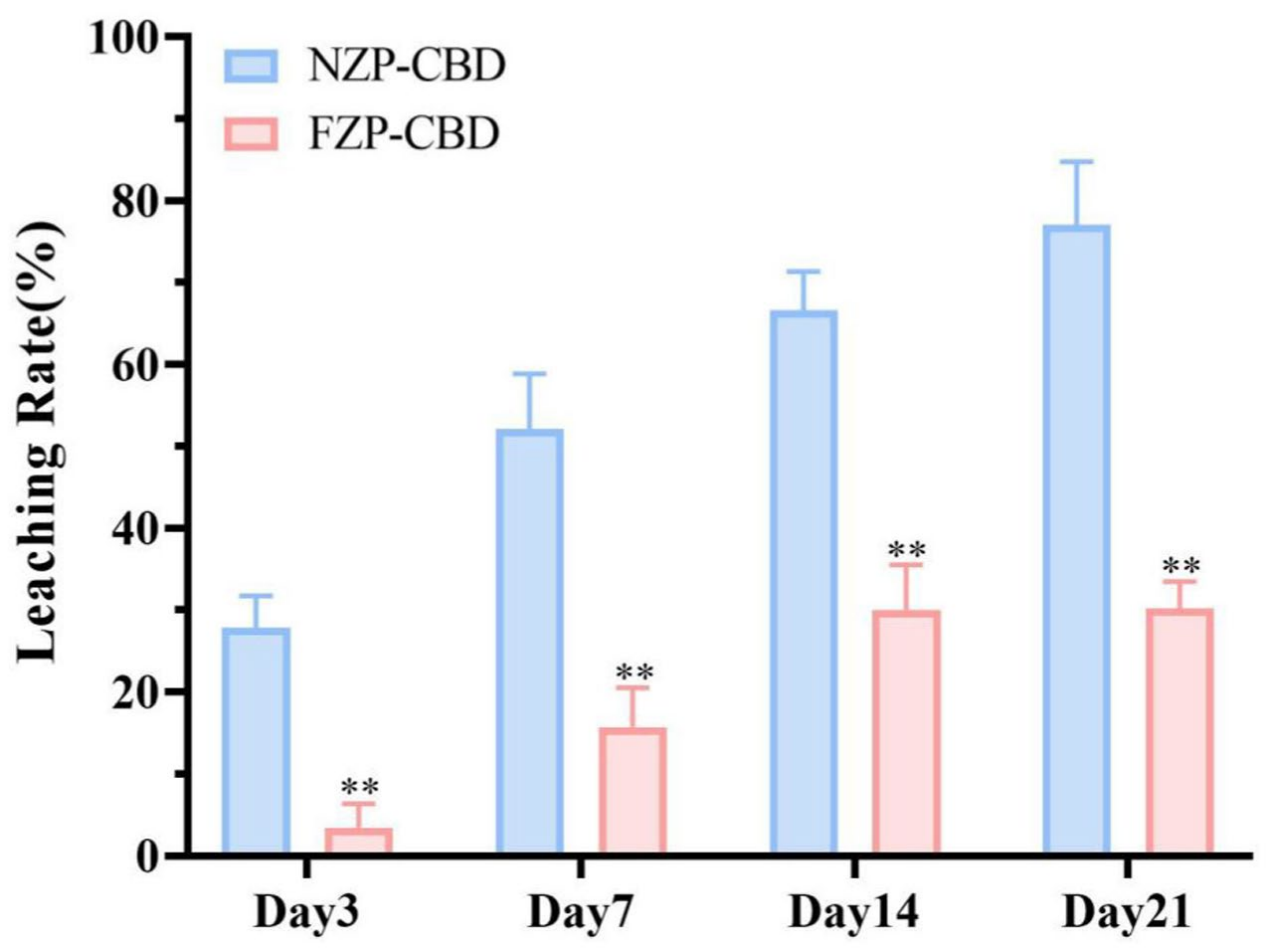
Storage stability of NZP-CBD and FZP-CBD. ^**^p < 0.01 compared to NZP-CBD (*x* ± s, *n* = 3).

The prepared NZP-CBD and FZP-CBD were stored at 4°C and protected from light. The degradation of CBD was measured on day 0, 3, 7, 14, and 21. Based on the leakage of CBD from nanoparticles in the medium after storage for 21 days, the storage stability was characterized by the leaching rate (as shown in Equation 3). The leaching rate of CBD in FZP-CBD was significantly lower than that of CBD in NZP-CBD throughout the storage period (*p* < 0.01) (Figure 3). It indicates that FZP-CBD improved the storage stability of CBD. This may be related to the ZP. Abdelbary et al. (39) concluded that the ZP was stable at around ±30 mV. The greater the absolute value, the better the stability. As can be seen in Figure 2C, the absolute value of FZP-CBD was significantly greater than that of NZP-CBD (*p* < 0.01), indicating that FZP-CBD has better storage stability.

### 3.5 Simulated gastrointestinal fluid release

**Figure 4:**
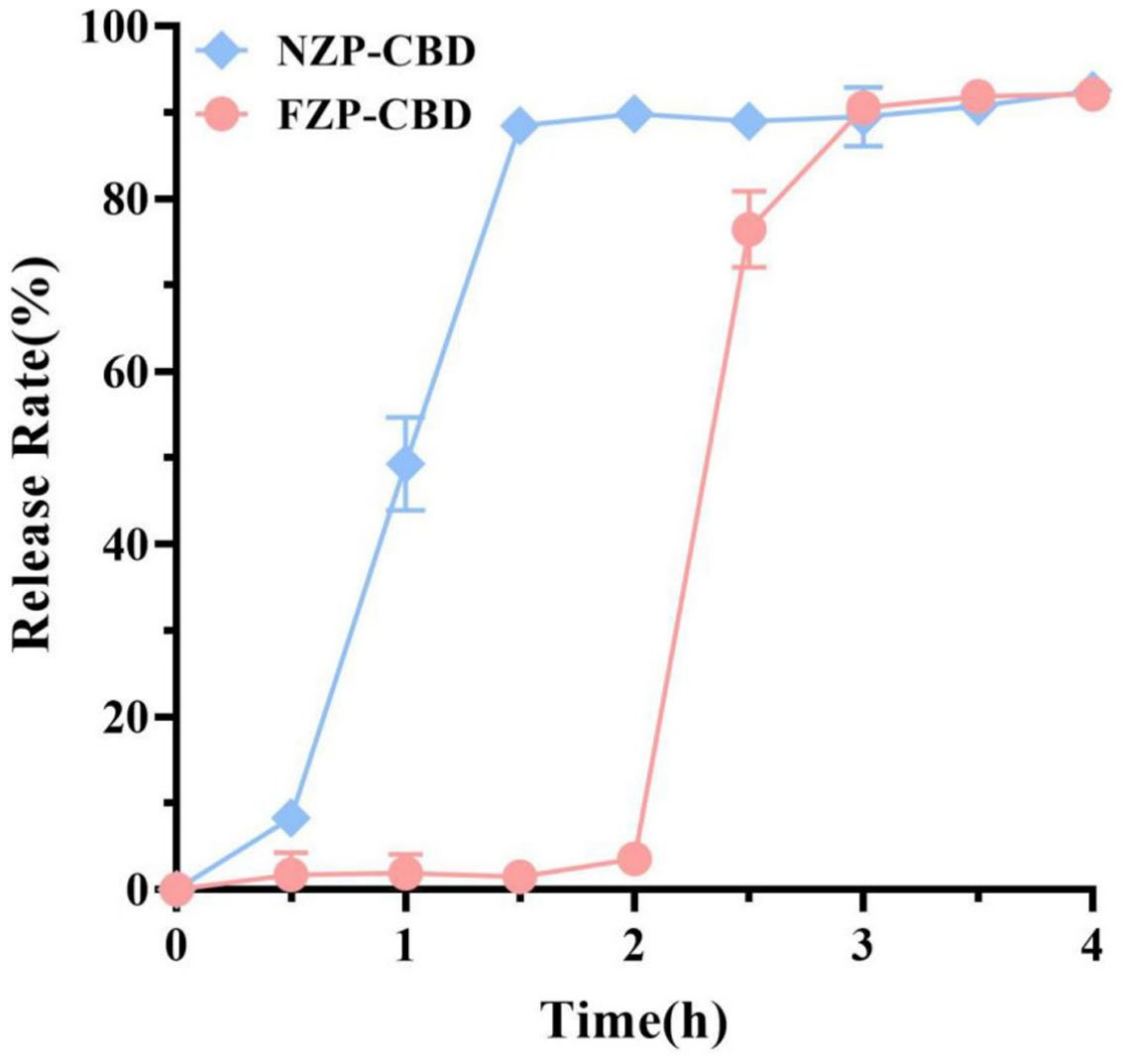
Release rates of NZP-CBD and FZP-CBD. 0–2 h was SGF, and 2–4 h was SIF.

The controlled release of CBD in the human gastrointestinal tract can play an important role in improving its bioavailability. In this study, the freshly prepared FZP-CBD and NZP-CBD were digested in SGF for 2 h and then transferred to SIF for further digestion for 2 h. The digestion was expressed in terms of the release rate. After digestion in SGF for 2 h, the release rates of FZP-CBD and NZP-CBD were 3.57 and 89.88%, respectively, which indicated that FZP-CBD had a protective effect on CBD in SGF. After digestion in SIF for 2 h, the release rates of CBD in FZP-CBD and NZP-CBD were 92.12 and 92.56%, respectively (Figure 4), indicating that both nanoparticles could fully release CBD in SIF, which may be due to the presence of pancreatin in the SIF (40). The results showed that FZP-CBD could protect most of the CBD from being released in the stomach, and then control its release in the intestines and promote the absorption of CBD in the small intestine, thus improving the bioavailability of CBD.

Pepsin causes structural changes in proteins by shearing the peptide bonds of hydrophobic amino acids. Meanwhile, deamidation decreases the hydrophobic amino acids and increases the hydrophilic amino acid content in protein molecules to protect proteins from destruction (32). Hurtado-López and Murdan (41) studied zein nanoparticles and showed that pepsin was able to digest α-zein monomers. The zein obtained via acid modification had decreased α-helix content and increased the β-fold content (33) so it was not easy to be digested by pepsin. CBD was encapsulated in the hydrophobic core of flexible zein, which was not easily destroyed by pepsin in SGF, and therefore only a small amount of CBD was released. When zein was transferred to the SIF, trypsin degraded the protein structure of flexible zein and CBD was released (22). This indicated that flexible zein could effectively avoid CBD leakage in gastric fluid. Due to the presence of pancreatin in the SIF (29, 41), FZP-CBD showed a sudden release effect during intestinal digestion. This results in the rapid decomposition of zein, which promotes the release and absorption of CBD and improves its bioavailability.

### 3.6 *In vitro* biocompatibility

**Figure 5:**
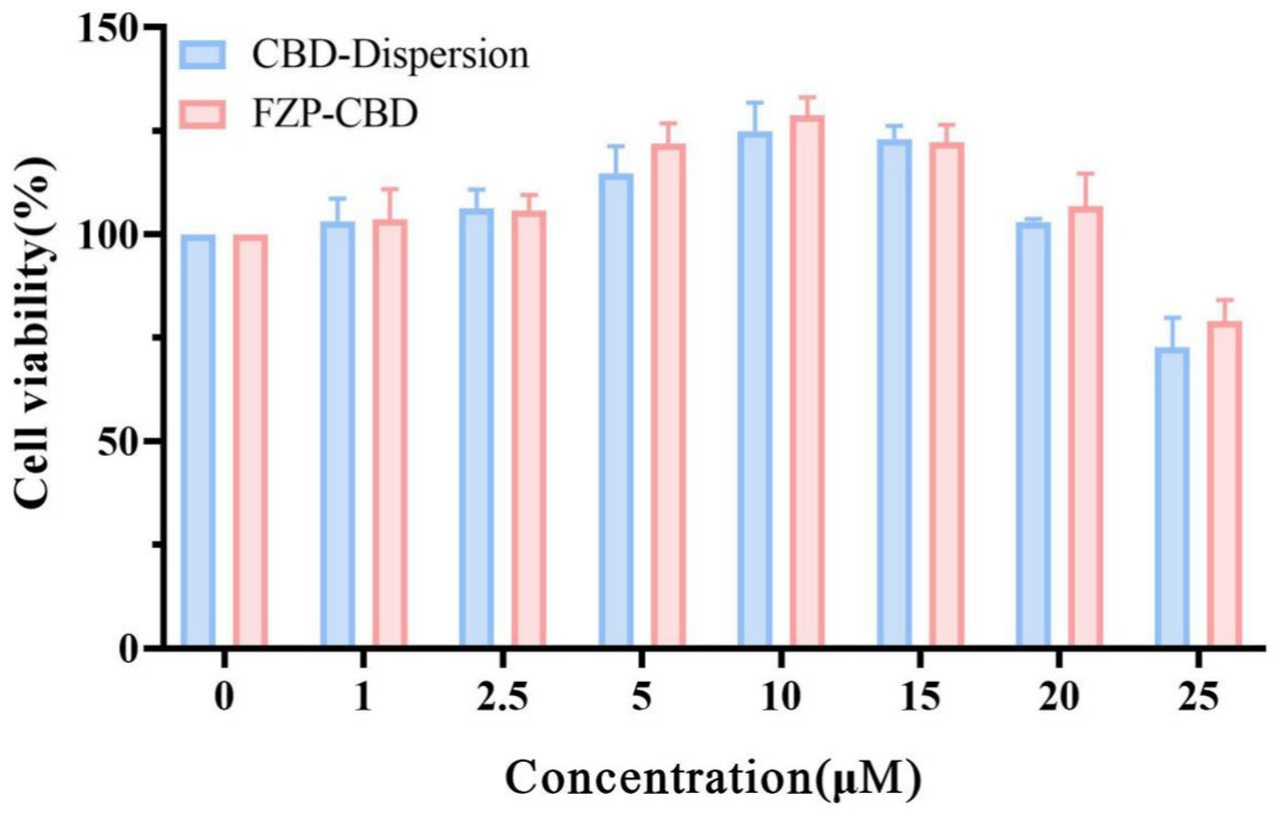
Cell viability of CBD-dispersion and FZP-CBD.

Using PC12 cells as *in vitro* cell lines, the cytotoxicity of CBD dispersion and FZP-CBD was studied by CCK8. CBD-Dispersion and FZP-CBD increased the viability of PC12 cells within the concentration range of 1–20 μM. However, when the concentration went beyond 20 μM and reached 25 μM, the cell viability of both CBD-Dispersion and FZP-CBD decreased and resulted in cytotoxicity (Figure 5). Therefore, within the safe concentration range, FZP-CBD does not produce toxicity to cells, indicating that flexible zein has good biocompatibility.

### 3.7 *In vivo* bioavailability

**Figure 6:**
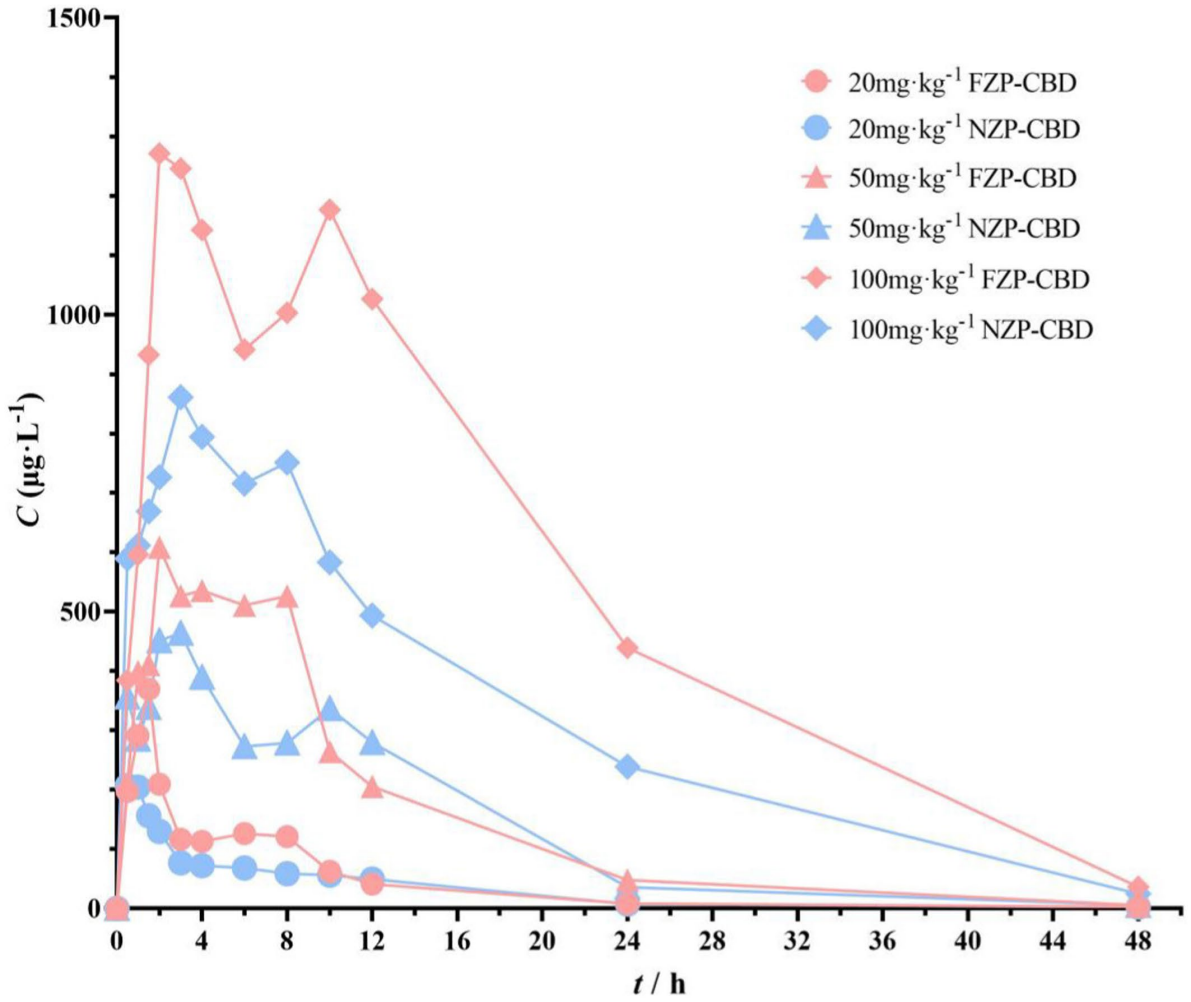
Serum concentration-time curves of NZP-CBD and FZP-CBD.

The pharmacokinetic differences of NZP-CBD and FZP-CBD in the serum of rats were observed and explored after a single intragastric administration. Compared with NZP-CBD, FZP-CBD improved CBD bioavailability (Figure 6). *C*_max_ of FZP-CBD at 3 different doses of CBD was increased by 1.7, 1.3, and 1.5 times respectively, and AUC*_0-t_* was increased by 1.4, 1.1, and 1.7 times respectively, bioavailability (F) was increased by 135.9, 114.9, 169.6%, respectively (Supplementary Table S3). Since CBD is a highly lipophilic drug, it is mainly absorbed in the small intestine. The results of the simulated gastrointestinal release experiments showed that FZP-CBD could protect most of the CBD from being released in the stomach, and then control its release in the intestine. Therefore, FZP-CBD could promote the absorption of CBD in the small intestine and improve CBD bioavailability.

## 4 Discussion

Currently, nano-delivery systems have been applied to CBD, including polymeric and lipid-based nanoparticles (lipid nanocapsules) (LNCs) (42), nanostructured lipid carriers (NLCs) (43, 44), nanoemulsions (NEs) (45) and self-emulsifying drug delivery systems (SEDDS) (46). These methods could improve the solubility and oral bioavailability of CBD to a certain extent, but there may be some problems in practical application. For example, lipid nanoparticulate drug delivery systems can improve drug stability. However, the high water content in the drug formulation may reduce the drug loading. In addition, the drug can easily leak due to lipid crystallization during storage (12). SEDDS is one of the most widely used encapsulation techniques of CBD with good stability and high oral bioavailability, but it contains high concentrations of surfactants which can potentially irritate the gastrointestinal tract (46).

In this experiment, we prepared zein nanoparticles to properly encapsulate and transport CBD. Encapsulating insoluble CBD in nanoparticles allows CBD to be well dispersed in water, which increases its solubility in the gastrointestinal tract and improves the passive transport of CBD across the epithelial cells of the small intestinal villi (47, 48). Nanoparticles can be absorbed by epithelial cells through endocytosis, and PS is the most important physical property that determines the endocytosis pathway (49). The preparation of nanoparticles in this study has a small size, which is more likely to be endocytosed by epithelial cells (50).

Furthermore, acid-modified zein was used to undergo a deamidation reaction, which improved the molecular flexibility. FZP-CBD could effectively improve the bioavailability of CBD and it has a more homogeneous particle size dispersion and higher ZP, EE%, LC% and stability compared with NZP-CBD. CBD possesses high lipophilicity. CBD can precipitate in the gastrointestinal (GI) tract when delivered orally in solution. The main reason for its low bioavailability is that it forms a precipitate in the stomach that is difficult to dissolve completely. Promisingly, acid modification decreased α-helix content and increased β-fold content in flexible zein thereby making it difficult to be digested by pepsin (33). In flexible zein, CBD was encapsulated in the hydrophobic core. Due to the resistance of flexible zein to pepsin, CBD exposure to gastric juice can be effectively prevented. When FZP-CBD reached the intestinal digestion stage, a burst release was observed potentially due to the presence of trypsin in the SIF (29). This enabled flexible zein to be rapidly decomposed which facilitated the release and absorption of CBD. The experimental results showed that FZP-CBD could protect most of the CBD from being released in the stomach, and then control its release in the intestines and promote the absorption of CBD in the small intestine, thus improving the bioavailability of CBD.

In conclusion, FZP-CBD provides a way to increase bioavailability by increasing the solubilization of CBD in the aqueous environment of the GI tract. The improvement in CBD bioactivity is not only due to the rapid decomposition in the intestine but also due to the delayed release: a combination of protection from gastric fluid and burst release in the intestine. This allows more CBD to cross the inter-villous spaces at the intestinal brush border and move into blood circulation.

## 5 Conclusion

In this study, the FZP-CBD could control the release of CBD in the intestinal tract and promote its absorption in the small intestine. It could improve the bioavailability of CBD. Therefore, this study can improve the utilization value of CBD and broaden the application scope of zein. Flexible zein can be used as a potential carrier of the highly lipophilic drug CBD, which provides a new idea to achieve the controlled release of highly lipophilic drugs in the intestine and the application of CBD in pharmaceutics.

## Data availability statement

The original contributions presented in the study are included in the article/Supplementary material, further inquiries can be directed to the corresponding author.

## Ethics statement

The animal studies were approved by the Animal Ethics Committee of Experimental Research Center, China Academy of Chinese Medical Sciences. The studies were conducted in accordance with the local legislation and institutional requirements. Written informed consent was obtained from the owners for the participation of their animals in this study.

## Author contributions

YK: Data curation, Formal analysis, Investigation, Writing – original draft. YN: Funding acquisition, Writing – review & editing. JP: Data curation, Formal analysis, Writing – review & editing. JS: Funding acquisition, Writing – review & editing. BF: Funding acquisition, Writing – review & editing.
