# Supplementary material for: Enhanced oral bioavailability of cannabidiol by flexible zein nanoparticles: in vitro and pharmacokinetic studies
Source: Front Nutr. 2024 Jul 17;11:1431620. doi: 10.3389/fnut.2024.1431620 (PMC11289775; doi:10.3389/fnut.2024.1431620)
Supplement: Supplementary file 1 [file Table_1.DOCX]

Supplementary Material

# Supplementary Tables

**Table S1.** Particle size (PS), polydispersity index (PDI) and zeta potential (ZP) of

NZP-CBD and FZP-CBD (*n*=3, *x*±s, ^*^*p*<0.05,^**^*p*<0.01 compared with NZP-CBD).

| Groups | PS (nm) | ZP (mV) | PDI |
| --- | --- | --- | --- |
| N-L | 111.60 ± 3.82 | 34.03 ± 1.43 | 0.17 ± 0.01 |
| F-L | 158.73 ± 3.83^**^ | -45.43 ± 2.79^**^ | 0.12 ± 0.01^*^ |
| N-M | 173.00 ± 9.49 | 36.99 ± 1.79 | 0.17 ± 0.02 |
| F-M | 198.07 ± 3.80^**^ | -45.53 ± 4.61^**^ | 0.15 ± 0.02 |
| N-H | 224.57 ± 6.30 | 38.10 ± 0.52 | 0.23 ± 0.04 |
| F-H | 203.93 ± 10.20^**^ | -51.34 ± 2.34^**^ | 0.20 ± 0.21 |

**Table S2.** Entrapment efficiency (EE%) and loading capacity (LC%) of NZP-CBD

and FZP-CBD (*n*=3, *x*±s, ^*^*p*<0.05,^**^*p*<0.01 compared with NZP-CBD).

| groups | *N* | | EE (%) | LC (%) |
| --- | --- | --- | --- | --- |
| N-L | 3 | 72.89 ± 5.55 | | 29.30 ± 5.38 |
| F-L | 3 | 92.23 ± 0.92 | | 34.68 ± 3.32^*^ |
| N-M | 3 | 62.11 ± 2.42 | | 36.86 ± 1.22 |
| F-M | 3 | 86.14 ± 2.57^*^ | | 48.86 ± 0.87^**^ |
| N-H | 3 | 79.27 ± 4.63 | | 53.63 ± 3.67 |
| F-H | 3 | 85.43 ± 3.71 | | 56.94 ± 2.47 |

**Table S3.** CBD related pharmacokinetic parameters (*n*=6, *x*±s, ^*^*p*<0.05,^**^*p*<0.01 compared with NZP-CBD).

| Parameter | 2mg·mL^-1^  NZP-CBD | 2mg·mL^-1^  FZP-CBD | 5mg·mL^-1^  NZP-CBD | 5mg·mL^-1^  FZP-CBD | 10mg·mL^-1^  NZP-CBD | 10mg·mL^-1^  FZP-CBD |
| --- | --- | --- | --- | --- | --- | --- |
| *T*_1/2_/h | 5.60 ± 1.65 | 9.04 ± 2.59 | 7.15 ± 1.17 | 5.91 ± 1.40 | 7.25 ± 2.07 | 8.93 ± 3.29 |
| *C*_max_/(μg·L^-1^） | 240.57 ± 143.79 | 414.78 ± 184.06 | 542.28 ± 235.37 | 715.29 ± 191.68 | 1173.30 ± 316.96 | 1798.44 ± 854.54^*^ |
| *T*_max_/h | 0.92 ± 0.38 | 2.42 ± 2.48 | 4.67 ± 5.04 | 4.25 ± 2.44 | 5.75 ± 3.82 | 5.33 ± 4.46 |
| AUC_0-_*_t_*/(h·μg·L^-1^） | 1438.64 ± 557.13 | \| 1955.24± 632.91 \| \| --- \| | 6316.54 ± 2916.26 | 7255.19 ± 1573.81 | 15674.15 ± 4365.67 | 26633.96 ± 16479.72^*^ |
| AUC_0-∝_/(h·μg·L^-1^） | 1443.03 ± 553.87 | 1989.33 ± 653.50 | 6375.13 ± 2924.86 | 7292.54 ± 1601.87 | 15877.51 ± 4524.75 | 27137.06 ± 16822.83^*^ |
| MRT_0-_*_t_*/h | 9.00 ± 1.88 | 7.51 ± 1.55 | 9.57 ± 1.23 | 9.16 ± 1.81 | 12.42 ± 2.37 | 13.12 ± 3.50 |
| MRT_0-∝_/h | 9.23 ± 1.95 | 8.34 ± 1.66 | 10.11 ± 1.30 | 9.38 ± 1.89 | 12.94 ± 2.63 | 14.07 ± 3.96 |
| F/% | - | 135.9 | - | 114.9 | - | 169.6 |
